# Understanding the implementation and effectiveness of a group-based early parenting intervention: a process evaluation protocol

**DOI:** 10.1186/s12913-016-1737-3

**Published:** 2016-09-15

**Authors:** Grainne Hickey, Sinead McGilloway, Mairead Furlong, Yvonne Leckey, Tracey Bywater, Michael Donnelly

**Affiliations:** 1 Mental Health and Social Research Unit, Maynooth University Department of Psychology, John Hume Building, National University of Ireland Maynooth, Maynooth, Co. Kildare Ireland; 2 UKCRC Centre of Excellence for Public Health, School of Medicine, Dentistry and Biomedical Sciences, Queen’s University Belfast, Belfast, Northern Ireland; 3 Department of Health Sciences, Faculty of Sciences, Seebohm Rowntree Building, University of York, York, UK

**Keywords:** Process evaluations, Program theory, Realist evaluation, Intervention fidelity, Early years intervention, Parenting programs, Mixed-methodology research

## Abstract

**Background:**

Group-based early parenting interventions delivered through community-based services may be a potentially effective means of promoting infant and family health and wellbeing. Process evaluations of these complex interventions provide vital information on how they work, as well as the conditions which shape and influence outcomes. This information is critical to decision makers and service providers who wish to embed prevention and early interventions in usual care settings. In this paper, a process evaluation protocol for an early years parenting intervention, the Parent and Infant (PIN) program, is described. This program combines a range of developmentally-appropriate supports, delivered in a single intervention process, for parents and infants (0–2 years) and aimed at enhancing parental competence, strengthening parent-infant relationships and improving infant wellbeing and adjustment.

**Methods:**

The process evaluation is embedded within a controlled trial and accompanying cost-effectiveness evaluation. Building from extant frameworks and evaluation methods, this paper presents a systematic approach to the process evaluation of the PIN program and its underlying change principles, the implementation of the program, the context of implementation and the change mechanisms which influence and shape parent and infant outcomes. We will use a multi-method strategy, including semi-structured interviews and group discussions with key stakeholders, documentary analysis and survey methodology.

**Discussion:**

The integration of innovations into existing early years systems and services is a challenging multifaceted undertaking. This process evaluation will make an important contribution to knowledge about the implementation of such programs, while also providing an example of how theory-based research can be embedded within the evaluation of community-based interventions. We discuss the strengths of the research, such as the adoption of a collaborative approach to data collection, while we also identify potential challenges, including capturing and assessing complex aspects of the intervention.

**Trial registration:**

ISRCTN17488830 (Date of registration: 27/11/15). This trial was retrospectively registered.

**Electronic supplementary material:**

The online version of this article (doi:10.1186/s12913-016-1737-3) contains supplementary material, which is available to authorized users.

## Background

The nature of the caregiving environment experienced at an early age plays a pivotal role in child development. Several studies and meta-analyses have demonstrated that early childhood interventions implemented between pre-birth and 3 years can help to improve parent-infant relationships and parenting skills [1–3]. Research has also demonstrated links between early intervention and improvements in child cognitive and language development [4, 5], as well as positive behavioral and socioemotional adjustment [6]. There is strong evidence, in particular, to support the effectiveness of home-visiting programs [7], while a smaller body of research points to the effectiveness of group-based early parenting programs for improving parental competency and infant adjustment [8, 9].

Recent significant advances in the delivery and testing of early parenting programs, as well as the accumulation of results from controlled trials, provide an important evidence base for ‘what works’ for parents and children [10]. Despite this progress, complex interventions are multifaceted in nature and their success and/or failure often depends on the context in which they are implemented. The results of controlled trials, however, provide little insight into how such interventions work or which components of complex interventions and contextual factors contribute to outcomes [11]. Indeed, many questions regarding the conditions, processes or program components which influence the effectiveness of early parenting interventions remain unanswered [12].

There is increasing recognition of the importance of effectively implementing prevention and early intervention programs in order to ensure positive outcomes [13, 14]. Indeed, a failure to do so has been shown to undermine the potential benefits for children and families [15, 16]. However, there is limited research about what constitutes effective implementation of early parenting interventions. Love and colleagues [6] demonstrated that parenting interventions established as part of the Early Head Start initiative produced a greater number of positive effects on parenting behavior, child cognitive development and socioemotional outcomes when implementers adhered closely to a set of specified performance standards. Regarding the implementation of home-visiting programs specifically, positive and trusting relationships between intervention providers and recipients [17, 18], as well as parental satisfaction and engagement with the intervention [19], have been found to be associated with program effectiveness. Higher program fidelity and parental engagement in group-based parenting programs have also been shown to be associated with better outcomes [20, 21]. A number of other studies have identified factors which are associated with the success of these programs including parental attitudes towards program content, changes in parenting skills and confidence, and positive experiences of the group process [22–24]. However, the sparse research in this area has focused only on the implementation of group parenting programs for parents of older children with conduct disordered behavior. Thus, little is known about how implementation components, processes and contexts of early parenting interventions, particularly group-based programs, influence outcomes for parents and infants.

The dearth of process-oriented research means that decision makers and service providers are often unclear about how and when such programs may be effectively embedded within community-based early years services [25, 26]. This protocol describes a process evaluation nested within a controlled trial of a group-based early parenting intervention program for parents and infants (aged 0–2 years). An accompanying economic evaluation will also be conducted. This large-scale, multi-site research program is designed to explore the development, implementation and effectiveness (and cost-effectiveness) of the program (see www.mhsru.com). The research program is being conducted over a 5 year period (2014–2019) [27]. This group-based, Parent and Infant (PIN) program aimed at improving child wellbeing (0–2 years) was developed in Ireland by an NGO called Archways in collaboration with Public Health Nurses (PHNs) and other community-based organisations. The program is an enhanced early intervention model which combines a range of group-based parenting supports and involves collaboration between multi-disciplinary stakeholders to tailor service delivery to infant, family and community needs, address multiple risk factors, tackle gaps in treatment and address barriers to engagement for ‘harder to reach’ families [28].

## Methods/Design

### Process evaluation aims and objectives

The overarching aims of this process evaluation are to gain an understanding of the active ingredients of the PIN program and to systematically evaluate the processes and conditions which influence program implementation and effectiveness. More specifically, the process evaluation will attempt to ‘unpack’ the program components in order to identify and describe the causal assumptions underpinning the program and, in turn, assess how those change principles are substantiated through implementation. The conditions/factors which influence or shape program implementation and responses to the intervention will also be examined. In this way, we will examine how the program works (or does not work) and whether there are facilitators and/or barriers to program implementation and effectiveness. The specific objectives (see also Table 1) are to:

1. *Develop a program theory*: Document, in detail, the design and development of the PIN program and outline its change principles and underlying assumptions.
2. *Assess implementation*: Outline the key resources involved in program implementation and the extent to which the program is implemented as intended (i.e. fidelity to the program model/change principles); examine patterns of participation within the program, including the role of program developers and implementers, as well as program reach, recruitment, and participants’ patterns of engagement and responses to the program; and identify implementation barriers/facilitators.
3. *Explore the context of program delivery*: Examine the conditions in which the PIN program is delivered including the individual, interpersonal and organisational capacities which support implementation; and explore the circumstances or contextual factors which influence the experiences of key stakeholders.
4. *Identify mechanisms of impact*: Identify patterns of interaction between the program, the stakeholders involved, and the context in which the program is delivered and explore the extent to which these shape, either positively or negatively, the implementation and impact of the program; and identify lessons for the development and implementation of other similar complex prevention and intervention programs.

**Table 1:** Process Evaluation Objectives and Research Questions

Objectives	Research questions
Developing a program theory	What are the components of the intervention? What are the causal assumptions underpinning the program? How is the program intended to be delivered?
Implementation	What resources are involved in program implementation? To what extent is the program implemented as intended over time and across settings? How do key stakeholders, including program developments and implementers and parent participants, participate in and respond to the intervention? Are there any barriers and challenges to program implementation?
Context	What are the characteristics of the service environment in which the PIN program is delivered? What are the broader conditional factors which impact on key stakeholders’ experiences?
Mechanisms of impact	How do program components, persons and contexts interact to influence program implementation and related outcomes? What generalisable lessons can be derived from the findings for the implementation of prevention and early intervention programs?

### Study design/process evaluation framework

A multimethod framework has been developed which draws on a range of process evaluation frameworks and theories, as well as mixed methodologies. Multimethod approaches are common in process evaluation research [35, 36] and can enhance the quality of the data/information generated, as well as the potential utility of the findings. While core aspects of the multi-component PIN program are standardised, other components are not; thus, there is likely to be innovation and adaptation of implementation across program sites and as program delivery proceeds. Moreover, complex interventions, such as the PIN program, are likely to act in a differentiated manner between persons and across contexts. The pluralistic approach adopted here will enable greater flexibility in data collection and analysis, as well as providing useful insights into implementation strategies and how they play out under different conditions and settings. This evaluation incorporates aspects of program theory [37] and realist evaluation [38], while also drawing upon implementation fidelity research [39] to address the study objectives. The development of this multimethod framework was informed by the 2014 Medical Research Council (MRC) framework for process evaluations [40].

Firstly, we will draw on program theory to document the assumptions underpinning the PIN program and outline a model of the way in which it is intended to work, whilst also highlighting the causal processes or change principles that are anticipated to lead to intended/expected outcomes (objective 1) [41]. The benefits of this approach lie in its ability to highlight the logic underpinning an intervention and, in turn, to build knowledge of how and why an intervention is expected to work [42, 43]. The approach also serves to forefront stakeholder perspectives and surface both underlying assumptions and intended implementation mechanisms. To date, the PIN program developers and implementers have not developed a systematic ‘model’ of the pathways, processes and activities involved in the PIN program and the ways in which these are assumed to influence outcomes. Thus, the ‘theory’ of the PIN program will be developed collaboratively through qualitative interviews, documentary analysis and liaison with key stakeholders. The development of a program theory will form an important foundation for this process evaluation.

The analysis of PIN program implementation (objective 2) will be guided by the work of Baronowski and Stables [39] who developed a framework for examining implementation fidelity. This framework highlights several priority areas for investigation and has been used to guide the development of supplemental research questions relevant to program implementation (Table 2). Some items, however, have been adapted or removed for improved fit with the PIN intervention and process evaluation objectives (e.g. context was removed from this framework as it already included in the overarching process evaluation research questions). This will enable us to explore facilitator adherence to program protocols, program delivery, reach and dosage and stakeholder responses to, and engagement with, the intervention. Thus, these findings will feed into an analysis of fidelity to the progam model, as well as the identification of barriers to/facilitators of program implementation and delivery.

**Table 2:** Framework for Documenting Implementation

Priority areas	Research questions	Data sources
(i) Recruitment	Who are the targets of the intervention? What processes are used to identify and recruit participants? What are the characteristics of parents who took part in the intervention?	▪ Documentation ▪ Liaison with program developers and implementers ▪ Impact evaluation data
(ii) Maintenance	How does the relationship between parent participants and implementers evolve over time? Which participants remain involved in the program over time and who withdraws from participation?	▪ Liaison with program developers and implementers ▪ Impact evaluation data ▪ Attendance sheets
(iii) Resources	What are the characteristics, materials and structures which support delivery of the intervention? What training, guidance and information do implementers receive? What structures and/or processes specify and direct implementation	▪ Interviews ▪ Stakeholder feedback ▪ Stakeholder survey
(iv) Implementation	To what extent was the intervention material delivered? How consistent is the delivery of the intervention? Were there planned or unplanned changes as the intervention is delivered? How does the involvement of implementers change over time?	▪ Stakeholder feedback ▪ Interviews
(v) Reach/Dosage	What percentage of the target participants attended the program? How well are the program components attended?	▪ Documentation ▪ Attendance sheets
(vi) Barriers	What problems are encountered reaching participants? What challenges to implementers experience in delivering the intervention?	▪ Interviews ▪ Group discussion
(vii) Responses to the intervention	How satisfied are key stakeholders with program components and the PIN intervention overall? What influences key stakeholders responses to the intervention?	▪ Stakeholder feedback ▪ Interviews ▪ Group discussion
(viii) Use	How do parent participants use the information/materials delivered as part of the intervention? Which aspects of the program are most useful for parents?	▪ Interviews
(ix) Continued use	Do parents continue to make use of program information/materials over time?	▪ Interviews
(x) Contamination	Do parent participants access additional services and supports? Do non-participating parents (i.e. control group parents) receive components of the intervention and/or other supports?	▪ Impact evaluation data

There is increasing recognition that programs can influence different people in different ways, while the success of implementation strategies and/or processes is frequently mediated by conditional or contextual influences [44]. Therefore, we will also explore the context in which the PIN program is delivered (objective 3) including:

- The service delivery context which will be conceptualised as incorporating the capacities of individuals (e.g. skills, attitudes, and motivation), organisational infrastructures (e.g. resources, funding, policies, roles and leadership) and interpersonal relationships (e.g. social capital and collaboration).
- The broader contextual factors which are external to the intervention and which may shape and/or influence participants’ responses to the intervention (e.g. parent/infant characteristics; socioculturally informed attitudes’ towards parenting; stakeholder’ attitudes towards the intervention).

We will explore, in turn, the interplay between the PIN program and its components, persons and conditions in order to gain insights into the processes of change arising from the intervention (objective 4). Thus, we will draw on a realist evaluation perspective [38] to investigate what works for whom, where, and under what circumstances, as well as identifying facilitative and/or inhibitive factors for program implementation and effectiveness. The benefits of realistic evaluation are that the approach focuses more specifically on the transformation process and why/how particular activities or intervention processes generate desired results, as well as highlighting the dynamic interaction between the specific context of program implementation and the mechanisms which shape intervention [45] outcomes.

**Fig. 1:**
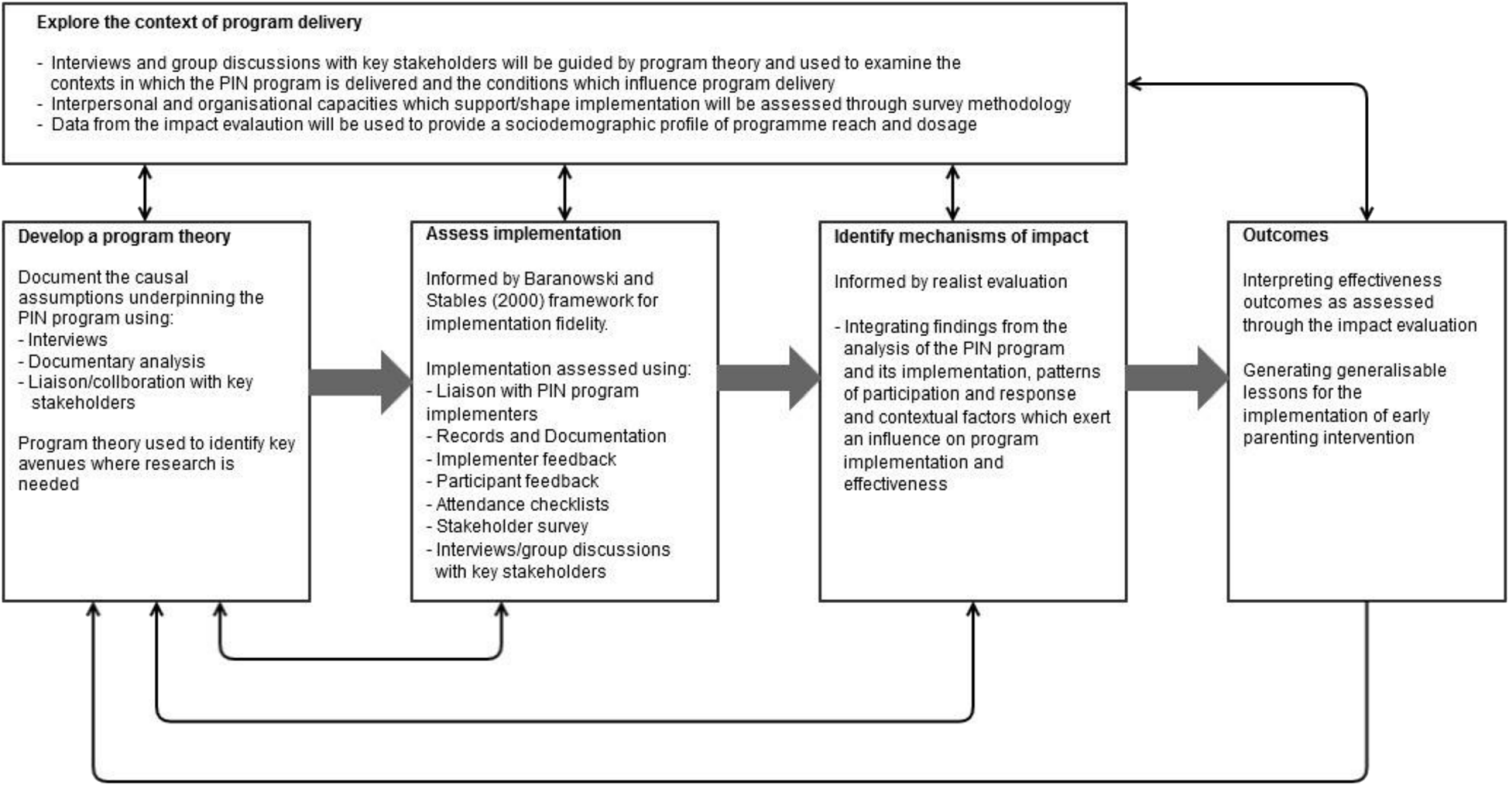
Framework for the process evaluation of the PIN program (informed by the MRC framework for process evaluations

Given the complexity of social interventions such as the PIN program, the program theory will be used to identify priority avenues for investigation in relation to the implementation and effectiveness of the intervention [46]. We will also draw on existing developmental theory, such as Bronfenbrenner’s [47] bioecological model of child development, to inform this work. According to this model, infants are agents in their own development, while complex processes within environments - such as patterns of reciprocal interaction between infants and parents - shape developmental outcomes [48]. The PIN program also draws on a number of parenting theories, such as behavioral and social learning theories, which emphasise the role of parenting in child development. Such theories, where relevant, will assist the prioritization of research questions and help to structure the analysis of how program components, implementation processes and contextual factors interact, thereby helping to identify change mechanisms and how they are triggered across different contexts (Fig. 1).

### Intervention

**Fig. 2:**
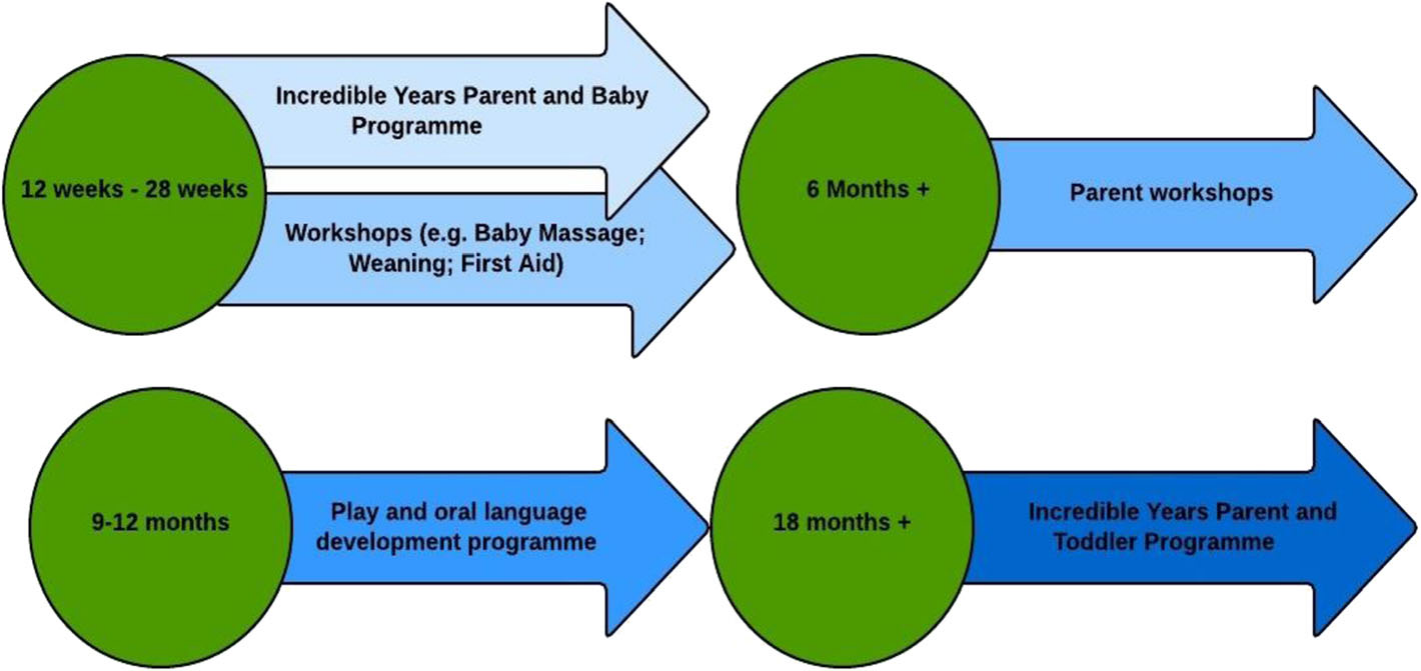
Outline of the Parent and Infant (PIN) Program

The PIN program combines a range of developmentally-appropriate parent and infant supports which are delivered in a single intervention process from birth to 2 years of age (Fig. 2). The program is designed to be flexible in the sense that content and delivery can be tailored to parent/community needs, but it also has standardised core elements including two newly developed Incredible Years (IY) parenting programs [29]. Thus, parents who have recently given birth are offered a 16-week IY Parent and Baby (IY-PBP) program straddling the first 6 months of development. During this period, the IY program is delivered on alternate weeks in conjunction with information and awareness-raising and practical workshops and classes for new mothers (e.g. baby massage classes, weaning workshops, paediatric first aid, dental health and child safety). Tailor-made play workshops, as well as oral language development supports are also offered to parents when the infant is between the 9–12 months old. Subsequently, when the child reaches 18 months, the Incredible Years Parent and Toddler Program (IY-PTP) will be delivered [30]. Both Incredible Years programs are rooted in behavioral and social learning principles; they use DVD modelling, group discussions and role play to help parents acquire new skills aimed at promoting positive child adjustment and preventing maladjusted behavior.

The goals of the PIN program are to: empower parents; strengthen parent competencies; build social support networks; enhance parent-infant relationships; encourage positive infant health and development and prevent injury; promote cognitive and pre-literacy skills; prevent conduct-disordered behavior; and enhance infant socioemotional development. The specific objectives of each program component are shown in Table 3.

**Table 3:** Parent and Infant Program Components, Core Topics and Objectives

Components	Core topics	Objectives
Incredible Years Parent and Baby program (8 sessions)	Getting to know your baby Babies as intelligent learners Providing physical, tactile and visual stimulation Parents learning to read babies’ minds Gaining support Babies’ emerging sense of self	▪ Strengthen parent knowledge and self-confidence through learning about babies’ development and developmental milestones ▪ Enhance parent-infant relationships and parental competencies, prevent infant maladjustment and promote infant wellbeing through skills and techniques to support learning and development and healthy behaviors (feeding, sleeping, calming babies) ▪ Empower parents through learning about self care and gaining support
Baby Massage (4 sessions)	Relief – Colic and wind; Emotional stress Relaxation – Soothes and aids sleep Stimulation – Build immunity and help gain weight Interaction – Aid bonding and reduce postnatal depression	▪ Enhance parent-infant bonding and alleviate infant stress ▪ Promote parental sense of competence and wellbeing (e.g. reduce postnatal depression)
Weaning workshop (1 session)	Stages of weaning, timing, quantities, feeding techniques Food safety and hygiene Healthy eating principles Practical cookery demonstration and advice	▪ Enhance parents’ knowledge/competencies in relation to healthy eating ▪ Increase healthy eating behaviors ▪ Prevent early weaning
Paediatric First Aid workshop (1 session)/Child safety† (1 session)	Child resuscitation Dealing with injury, poisoning, choking and medical emergencies Recovery position Threats to child safety and child proofing home environments	▪ Prevent/Reduce incidents of injury to infants through parents learning first aid skills and baby-proofing home/environments techniques; and ▪ Enable parents (promote sense of competence) to identify, remove and respond to threats
Dental health† (1 session)	Principles of dental health	▪ Increase parents’ awareness of oral health ▪ Improve parents’ knowledge/competencies in relation to oral health
Toddler Healthy Eating^a^ (1 session)	Food safety and hygiene Healthy eating principles Practical cookery demonstration and advice	▪ Enhance parents’ knowledge/competencies in relation to healthy eating ▪ Increase healthy eating behaviors
Returning to work workshop (1 session)	Information on childcare options Guidelines for choosing childcare	▪ Empower parents/reduce parental anxiety in relation to returning to work
Active Play† (2 sessions)/ Play & Oral Language Development program^a^ (4 sessions)	Play skills and strategies Language development milestones Practical play sessions and advice	▪ Strengthen parent knowledge and competencies through playing skills and strategies ▪ Enhance parent–child relationships and encourage child wellbeing through play ▪ Promote child language development and pre-literacy skills
Incredible Years Parent and Toddler Program (8 sessions)	Child directed play promotes positive relationships Promoting toddler’s language with child directed coaching Social and Emotion coaching The art of praise and encouragement Spontaneous incentives for toddlers Handling separations and reunions Positive discipline – effective limit setting Positive discipline – handling misbehavior	▪ Strengthen parent knowledge and self-confidence through learning about toddler development ▪ Enhance parent-infant relationships and parental competencies, prevent child maladjustment and promote socioemotional wellbeing (e.g. self-regulation and self-esteem) through building parent’ coaching/modelling skills and play skills and strategies ▪ Promote child language development and pre-literacy skills

†Delivered in Site 2 Drogheda/Dundalk only

^a^Delivered in Site 1 West Dublin only

### Study setting

The program is being implemented in two separate sites in the Republic of Ireland: West Dublin and Drogheda/Dundalk, Co. Louth^1^ (Northeast Ireland). Both are urban areas characterised by significant socioeconomic disadvantage and outcomes for children and young people in these areas compare unfavourably to national averages [31, 32]. The program will be delivered to groups of parents over two to three^2^ cycles per year. Each cycle comprises 2–3 parent groups comprising approximately 8–10 parents per group. Program delivery began in January 2015 in Site 1 (West Dublin) and roll-out in Site 2 (Drogheda/Dundalk) commenced in September 2015. The implementation of the PIN program is currently funded for a 2 year period (2014–2016) and five to six cycles of program delivery will be initiated during this period, although it is anticipated that program delivery will continue beyond this time point.

The delivery of the PIN program is funded jointly by the Irish government and a philanthropic organisation called the Atlantic Philanthropies, as part of a new Area-Based Childhood (ABC) program in Ireland [33]. The ABC program involves the implementation of 13 area-based approaches/initiatives to preventing and reducing child poverty in socially deprived areas of Ireland; two of these - the Blue Skies Initiative in Site 1 and the Genesis Program in Site 2 - are currently delivering the PIN program as one of a number of services in their area-based approaches. In each Site, the PIN program has been customised to meet community needs and local service delivery capacities. Thus, there are minor differences in the content and process of delivery between Site 1 and Site 2 (see Table 3) which will be explored through this process evaluation. The development and implementation of the PIN program is overseen in each site by a multi-disciplinary consortium of local stakeholders. These consortia employ a small number of staff whose role is to support participant recruitment and engagement, as well as service delivery. The program is delivered via usual care services and agencies, including statutory health care services (i.e. Public Health Nursing) and community-based organisations.

### Evaluation of the PIN program

A detailed process evaluation is being conducted in conjunction with a large-scale, longitudinal controlled before-and-after impact evaluation to assess the effectiveness of the program. Parents from within the sites where the program is delivered are recruited to the PIN program by Public Health Nurses and/or local, community-based service providers. Parents who are recruited to the program will subsequently be invited to participate in the research. A comparison group of parents who receive services as usual (and no PIN intervention) will be recruited from neighbouring areas with a similar profile to the intervention areas. Usual services in the non-intervention area for parent-infant dyads involve: one home visit from a PHN in the first 6 weeks after birth, regular developmental check-ups with a GP/PHN and vaccinations.

Prospective participants will be informed of the research verbally and by means of a brief brochure describing the research. Potential participants will then be asked to provide their written consent to be considered for inclusion in the research and for their details to be passed to the research team. If this consent is provided, names and telephone numbers are confidentially passed to the research team. Participants will then be contacted by the research team and furnished with a detailed information sheet. All participants must be aged 16 years or older and will provide written informed consent. A power calculation conducted on the basis of comparing the mean score of an intervention group on the Parenting Sense of Competence Scale (PSOC) [34] to that of a control group of the same size. Data will be required from 132 parents (66 in the intervention group; 66 in the control group) to allow over 80 % power to detect a difference in mean PSOC scores, between the intervention and control arm, of 3 units, based upon a SD of 6, this corresponds to a Cohen’s d of 0.5 (medium effect size). Therefore, a total sample size of 200 parents was recommended, assuming an attrition rate of 33 %.

Baseline assessments will be conducted with parents when the infant is approximately 6 to 14 weeks old. Subsequent follow-up assessments will be carried out when the infant is approximately 8, 16 and 24 months of age. The impact of the program, when compared to services as usual, will be investigated by assessing parent competencies and wellbeing, parent-infant relationships, child development and socioemotional adjustment. The process evaluation will be vital to interpreting the outcomes of the PIN program, while it also forms part of a wider goal to contribute to our understanding and knowledge of the effective implementation of complex early parenting intervention programs, including the relationships between program components, implementation processes, contextual factors and program outcomes. The process evaluation is being conducted by a multi-disciplinary research team independent of program development, implementation and delivery.

### Study participants and data collection

Study participants will include program developers and implementers, as well as parents who take part in the PIN program. This process evaluation will be based on both qualitative and quantitative methods. Data collection will be conducted in two focused phases. During the first (initial) phase, data collection will focus on explicating the theory underlying the intervention, identifying the causal assumptions inherent in the program and outlining the activities, resources and factors which are assumed to be necessary to achieving program outcomes.

The second phase of data collection will expand on early findings and focus on exploring the processes involved in program implementation, patterns of engagement with the program, as well as the context in which the program is situated. Both quantitative and qualitative data will be gathered to explore program delivery processes, uptake and coverage, responses to the intervention, barriers to effective implementation of the program (e.g. program reach and participant engagement) and competing programs. As the PIN program is already being implemented, quantitative data relevant to program implementation (e.g. attendance sheets, participant feedback forms, facilitator feedback forms) will also be collected during phase one of data collection. An in-depth examination of program implementation and contextual factors will inform a critique of fidelity to the PIN program theory, while also highlighting adaptation/innovation and enabling the interpretation of findings from the impact evaluation. The data gathered throughout the process evaluation will be synthesised and analysed in order to identify the mechanisms through which the intervention activities produce intended or unintended effects.

#### Data sources

A wide range of data sources will be used to develop an in-depth understanding of the PIN program, the key activities, processes, inputs and outputs involved in, and conditions associated with, program delivery and implementation.

*Phase 1 data sources*

1. Liaison with PIN program developers and implementers: The evaluation team and program developers and implementers are in regular communication. This will provide important insights into program logic, the choices made in relation to program content, preparation and groundwork for delivery as well as the planning and management of implementation.
2. Documentation: A documentary analysis will be carried out which will involve the review and critical analysis of a wide range of relevant documentation including: meeting minutes; organisational reports and publications; online material; brochures; program proposals and descriptions; program manuals and written materials; official policy documents and demographic data; publications, reports, and journal articles. This data will contribute to refining ideas and identifying the conceptual foundations of the PIN program.
3. Interviews: Program developers and implementers will be interviewed to explore the developmental origins of the PIN program, factors which influenced intervention design, development, and content, as well as related motivations, attitudes, perceptions and beliefs.

*Phase 2 data sources*

1. Liaison with PIN implementers: Regular contact/meetings between the PIN program Project Coordinators (in both Site 1 and 2) and the evaluation team will be held to provide updates on: the development and implementation of program components; choices made in relation to program content and implementation; participant recruitment; the timing, delivery and implementation of intervention components; implementation and operational activities; as well as any planned and/or unplanned or forced changes to the program components and its delivery. Regular contact will also enable the evaluation team to monitor how operational and implementation activities evolve over time.
2. Records and documentation: Records of recruitment procedures will be taken, including the number of prospective participants invited to participate in the program. Recruitment protocols, implementation manuals and other relevant documents (e.g. meeting minutes) will also be included in a documentary analysis. Demographic data, as well as routine documentation maintained by participating organisations (e.g. PHNs) including area birth rates, will be used to critique program reach.
3. Impact evaluation data: Participant demographic data collected through the impact evaluation baseline assessments will be used to examine the characteristics of parents who engage with, and continue to participate in, the program. This data will be used to assess program reach and the maintenance of participant engagement. All parents who take part in the trial will also be asked, by means of a follow-up questionnaire administered by the evaluation team, to report uptake of services and supports which are external to the PIN intervention. In addition, parents in the control group will asked if they have accessed any of the treatment components (e.g. Baby Massage). This will enable the research team to examine if, and to what extent, contamination is occurring, thereby facilitating the accurate interpretation of program outcomes.
4. Attendance checklists: Parent participants attendance for each program component will be routinely recorded by program facilitators. The number of sessions per component which individual parents attend, as well as the attendance of partners (if applicable), will also be recorded. Reasons for parental absence/non-attendance will be documented if available.
5. Parent participant feedback forms: Parent feedback forms will be administered following the completion of each program component. These questionnaires will measure participant satisfaction as well as the perceived utility of the content of each program component, and facilitator effectiveness (Additional file 1).
6. Implementer feedback forms: Program staff and facilitators will complete checklists/feedback forms after program sessions to record the extent to which essential material is covered, as well as the perceived response of participants. These forms will provide a measure of the extent to which the program is implemented as intended (fidelity); as well as highlighting any deviations from program content/protocols (Additional files 2, 3 and 4).
7. Stakeholder survey: Key stakeholders involved in program development, implementation and delivery will be asked to complete a questionnaire to examine stakeholder attitudes and perceptions towards the PIN program, as well as individual capacity (e.g. skills, capacities, motivations and attitudes) and organisational capacity building processes (e.g. availability of training and/or other supports for program implementers) (Additional file 5). Findings from the questionnaire will help to build an understanding of the program delivery context.
8. Interviews and group discussions: A series of one-to-one semi-structured interviews and group discussions will be conducted with a range of stakeholders as outlined below (Additional files 6 and 7). (a) Interviews will be conducted with program implementers (e.g. program support staff, facilitators) and management personnel from participating organisations; these will explore experiences of program implementation, barriers and challenges to implementation and the conditions within which the program is delivered (e.g. motivations, attitudes, perspectives, organisational infrastructures, policies, etc.). Group discussions will be conducted at a later stage and will focus on key stakeholders’ perceptions of how program implementation is progressing, any changes to program implementation, responses to environmental barriers and challenges and contextual changes (e.g. organisational changes).
  (b) Parent participants will be interviewed to explore issues pertaining to parenthood, including the experience of becoming a parent, parental expectations and the experiences and challenges/difficulties of parenting during infancy. These interviews will also focus on: parents’ experiences of taking part in the program; the extent to which they use the parenting skills, strategies, materials and information specified in the program; whether they continue to use such skills, strategies, material and information over time; barriers to program engagement/attendance; and factors which influence parenting experiences. A small number of partners of participating parents may also be invited to take part in qualitative interviews.

*Interview/group discussion procedure and participant sampling*. All interviews and group discussions will be audio-recorded with consent and guided using a schedule of open-ended questions. Interview/group discussion questions will be altered to reflect stakeholders’ experiences in relation the PIN and stages of program implementation (e.g. to explore emergent issues in implementation and/or participation in the PIN program). A purposive sampling method will be used to identify and recruit participants. Selection and recruitment will be designed to include as wide and diverse a range of perspectives as possible and to access participants’ expertise in relation to the key research questions. Key inclusion/selection criteria include the participants’ role within the development and/or delivery of the PIN program and their ability to provide insight into the key issues influencing, and affected by, program development and implementation. Parent participants will be selected from the overarching intervention group recruited to take part in the PIN program and the effectiveness evaluation (*n* = 100 approximately). Key demographic variables (e.g. socioeconomic disadvantage, marital status, age, parity, gender of child), program delivery cycle and level of program engagement (e.g. no. of sessions attended) will be used to recruit parents to the process evaluation. Care will be taken to ensure diversity and balance throughout the sampling process.

It is anticipated that approximately 40 key stakeholders will be invited to take part in interviews/group discussions. A responsive approach to sampling will be adopted; thus, as the research and data collection process progresses, potential participants will be identified and recruited on the basis of emergent findings. During the later stages of data collection, a small number of key informants will be re-interviewed or invited to take part in groups discussions in order to assess any potential changes over time in program implementation and delivery context, as well participants’ experiences, attitudes and beliefs.

### Data management and analysis

#### Qualitative data

All qualitative data will be retained as written electronic files including document summaries and interview and group discussion transcripts. Interviews and group discussions will be recorded (with consent) and transcribed verbatim. Audio-files will be destroyed after transcription and all qualitative data will be stored securely on a password protected computer. All transcripts will be anonymised and any potentially identifiable data removed.

A thematic analysis will be used to systematically analyse all qualitative data [49]. This analytical technique will involve five key stages: familiarisation, defining themes, coding, charting and interpretation. The familiarisation stage will involve an in-depth reading of the data and the generation of detailed summaries; subsequently codes will be developed by interrogating the data and linking narrative content to larger, more general processes or categories which capture the meaning of the data. Later stages of analysis will involve the categorisation of codes into overarching themes, finalising conclusions and interpretations, while also determining the strength and depth of the findings. This process will be supported by the use of MAXQDA. Reporting of qualitative findings will adhere to the consolidated criteria for reporting qualitative research (COREQ) [50].

#### Quantitative data

Quantitative data from implementer and participant feedback forms, attendance checklists and questionnaires will be entered into a password protected database and will be analysed in SPSS using appropriate descriptive and inferential statistics. Analysis of the stakeholder survey will focus on variability across groups, while the extent to which the intervention is delivered as intended will be examined by exploring the proportion of the essential material which was reported as delivered. Variability in the extent to which the program is delivered as intended across facilitators/parent groups and change over time will also be examined. In addition, the proportion of parent participants who report high levels of satisfaction with, and high perceived utility of, the PIN program and its sub-components will be reported. We will also explore whether there are any significant differences in satisfaction and perceived utility across parent participant sub-groups (e.g. primiparous and multiparous mothers). Program reach will be assessed by examining the proportion of the mothers who have given birth in the treatment areas and who are recruited to, and engage with, the program. Dosage will be analysed by determining the average number of program sessions attended by parent participants. Systematic differences between those who attend a high percentage of sessions and those who fail to engage/attend a low number of sessions will also be assessed.

#### Synthesising data

The blending of findings from different and multiple data sources will be an important step in refining the interpretations of program outcomes, understanding the context of program delivery and, in particular, identifying mechanisms of impact. For example, narrative accounts of implementation (e.g. transcribed interviews) will be triangulated with findings from the stakeholder survey and implementer/participant feedback forms, as well as outcome data from the impact evaluation. At this stage of analysis, data will be interrogated in order to identify patterns of interactions between the PIN program theory and its implementation, participant responses and contextual factors. Thus, the data will be appraised in order to understand how the PIN program works and, in turn, to identify key mechanisms which are critical to program success or, conversely, where program and/or implementation failures are likely to occur.

#### Rigor

This process evaluation will be carried out by a small team of experienced researchers from different backgrounds with considerable experience both of mixed methods and of undertaking large-scale community-based evaluations. A number of steps will be undertaken to ensure a rigorous approach to data collection and analysis including: (i) a purposive and responsive sampling method to ensure a broad and diverse participant sample and, in turn, good conceptual generalisability; (ii) a systematic approach to data collection and analysis and drawing on a rich range of data sources including quantitative and qualitative data; (iii) triangulation of findings across multiple sources of data; (iv) respondent validation via reiterative sampling and transcript reviews; (v) reflexively assessing the role and input of the researcher(s) with respect to data generation and data analysis; and (vi) ‘sensitivity to context’, including reference to appropriate literature and theory, as well recognising and examining differing perspectives and the context in which data and findings are generated [51]. Thus, participants’ expertise, group memberships and relationships to the PIN program represent important theoretical considerations. Documents and narratives cannot be considered independent or objective accounts of a phenomenon, process or event; rather they represent particular values and ideas [52]. Embedding this form of reflexivity into the analysis is vital to ensure the transferability of the findings.

## Discussion

In this paper we provide a detailed protocol for a process evaluation nested within a controlled trial of an early parenting intervention program aimed at improving parental competencies and infant wellbeing in the earliest years of life. It is increasingly recognised that implementation is vital to the effectiveness of prevention and early intervention programs [53]. New programs and practices, evidence-based or otherwise, cannot be simply transported into usual care services for children and their families [54]; rather, integrating innovations into existing systems is a challenging multifaceted undertaking. Thus, exploring and understanding what works for whom and what constitutes effective implementation, is an important consideration for researchers, practitioners and policy makers alike [55, 56]. This process evaluation will contribute invaluable knowledge, insights and understanding in relation to the implementation of a group-based early parenting program, while also providing a useful example of how process-oriented research can be embedded within the evaluation of community-based interventions.

### Strengths and challenges

This evaluation will adopt a comprehensive and rigorous, approach to the evaluation of the structures, processes and causal mechanisms inherent in the implementation of the PIN program. Nuanced information regarding the attitudes, characteristics and skills of program implementers, as well as interpersonal interactions, resources and circumstances around program implementation, will be collected using a broad range of quantitative and qualitative research methods.

This research will also be conducted by an experienced, multidisciplinary team of researchers who are working in close collaboration with a range of community-based stakeholders involved in program delivery and implementation. Positive working relationships between the evaluation team and program developers and implementers have already been developed and are being closely monitored and maintained. This will be important in supporting the process evaluation in a number of ways. For example, regular positive communication between the research team and stakeholders will ensure that the evaluators are notified and informed about any planned phases of implementation. Challenges and changes to program content and delivery can also be highlighted. In addition, PIN program implementers will assist with data collection (e.g. maintaining and providing participant feedback forms) and are also committed to monitoring fidelity and implementation processes. While this will help to reduce the costs of data collection and participant burden, facilitator self-assessment of fidelity and distribution of participant evaluation forms may bias implementation and/or result in Hawthorne effects. However, similar procedures for implementation fidelity monitoring have been reported elsewhere [57, 58]. Furthermore, if the PIN program (if found to be effective) is to be ‘scaled up’ and integrated into existing early years care services and systems, implementation fidelity and parent engagement should be routinely monitored by facilitators/implementers. Thus, such procedures can be considered characteristic of essential implementation practices. High quality data collection processes will be supported by regular team meetings and a focus on researcher reflexivity.

Potential challenges must also be noted. Firstly, the evaluation team will not communicate emerging findings from the process evaluation to key stakeholders in order to avoid any interference in the implementation process. However, the PIN program theory will be developed in collaboration with key stakeholders, whilst an interim evaluation report will also be made available prior to the completion of this study. There may also be instances where it is ethically imperative to communicate findings of the process evaluation to program developers/implementers (e.g. evidence of harm). It is possible that such interactions between the evaluation team and program developers/implementers may influence the development of the program and/or implementation activities. Secondly, the team of researchers with responsibility for conducting this process evaluation are also involved in conducting the outcome evaluation and cost-effectiveness analysis. This integration will be important in facilitating data sharing and in minimising participant burden; however, data collectors cannot be blind to program allocation and, therefore, some potential for bias in the interpretation of program functioning and outcomes may arise. For this reason, a reflexive approach to data collection and analysis will be adopted in order to minimise any bias and improve the reliability and validity of the findings. Analyses of program effectiveness will be conducted by the research team’s Data Manager who is blind to group allocation and not involved in any way in data collection.

Finally, the complex nature of the PIN program presents challenges. For example, the program consists of multiple interrelated and interdependent components, as well as multiple stakeholders. Some elements of the program are manualised (e.g. IY programs), while other components are not. Additionally, there are differences in program content and implementation processes across sites, while there is also likely to be adaptation and learning by those implementing and those receiving the intervention as delivery proceeds [59, 60]. The theory-oriented framework adopted here is designed to be responsive to the multifaceted nature of the intervention (e.g. use of a multi-method design and extensive data collection) and is aimed at capturing the multiple and/or alternative causal processes which may be associated with program outcomes (e.g. assessing the interactions between program components, people and conditions and how they relate to program outcomes). However, there remain significant challenges in monitoring and precisely assessing implementation processes and how they relate to outcomes vis-a-vis program delivery/implementation adaptation. Furthermore, the context in which the program is being implemented cannot be considered static, while it is also important to note that the relationship between context and implementation is not unidirectional. Thus, not only is the program being implemented within shifting environments, but the ongoing embedding of the program may also exert some influence on the environment. Thus, ‘pinning down’ the effects of, and interactions between, various program components and changing environments is not a trivial task.

## Conclusion

There remain significant barriers to ensuring that vulnerable children and families receive high-quality early intervention and prevention services and supports [61]. It is imperative, in the context of an increasing commitment to public investment in early years services, that methodologically rigorous, high quality research is conducted to help articulate how such services may be usefully embedded within mainstream service settings. For this reason, more high quality process evaluations are required to build a holistic understanding of what works in terms of prevention and early intervention, as well as what factors and conditions facilitate or inhibit the effectiveness of services for young children and their families. Preliminary findings from this research should be available in 2016. A final report, combining findings from the outcomes trial, cost-effectiveness study and process evaluation, will be published in 2018.

## Additional files

Additional file 1: Example participant feedback form (for workshops). (DOCX 64 kb) Additional file 2: The Incredible Years Parent and Baby Program Parent Satisfaction Questionnaire. (PDF 402 kb) Additional file 3: The Incredible Years Parent and Toddler Program Parent Satisfaction Questionnaire. (PDF 54 kb) Additional file 4: Example implementer/facilitator feedback form. (DOCX 13 kb) Additional file 5: Example stakeholder survey questionnaire. (DOCX 21 kb) Additional file 6: Example qualitative interview schedule with parents. (DOCX 15 kb) Additional file 7: Example qualitative interview schedule with implementers. (DOCX 16 kb)

## Abbreviations

ABC: Area-Based Childhood
IY: Incredible Years
IY-PBP: Incredible Years Parent and Baby Program
IY-PTP: Incredible Years Parent and Toddler Program
MRC: Medical Research Council
PHN: Public Health Nurse
PIN: Parent and Infant
PSOC: Parenting Sense Of Competence

## Footnotes

1 The Parent and Infant Program is known as the ‘Up to 2’ Program in West Dublin and the ‘Parent and Baby’ Program in Drogheda/Dundalk.

2 Three cycles per year in West Dublin and 2 cycles per year in Drogheda/Dundalk.
