# Supplementary material for: Understanding the implementation and effectiveness of a group-based early parenting intervention: a process evaluation protocol
Source: BMC Health Serv Res. 2016 Sep 15;16:490. doi: 10.1186/s12913-016-1737-3 (PMC5025622; doi:10.1186/s12913-016-1737-3)
Supplement: Additional file 1: — Example participant feedback form (for workshops). (DOCX 64 kb) [file 12913_2016_1737_MOESM1_ESM.docx]

***As part of the ENRICH research programme, we would like to get
your views on the programme you are attending and would appreciate if
you could complete the short form below. Thank you for your help.***

**NAME (optional):** ................................

**1. Why did you attend this session?**

…………………………………………………………………………………………………………

…………………………………………………………………………………………………………

…………………………………………………………………………………………………………

**2. How useful did you feel the session was for you *(circle number)*?**

| **5** (very useful) | **4** | **3** | **2** | **1** (not useful) |
| --- | --- | --- | --- | --- |

**3. What are the most useful ideas or techniques you learnt?**

…………………………………………………………………………………………………………...

…………………………………………………………………………………………………………...

…………………………………………………………………………………………………………...

**4. In general, was the session (choose one option)**

**4a)** Too long **🞏 Because:______________________________________________________**

**4b)** About right **🞏**

**4c)** Too short **🞏 Because:______________________________________________________**

**5. How would you rate the session materials/handouts *(circle number)*?**

| **5** (very useful) | **4** | **3** | **2** | **1** (not useful) |
| --- | --- | --- | --- | --- |

**6. Is there anything else that could be included in the session and if so, why?**

………………………………………………………………………………………………………...

………………………………………………………………………………………………………...

**7. Is there anything that could be left out of the session and if so, why?**

………………………………………………………………………………………………………...

………………………………………………………………………………………………………...

**8. How effective did you feel the facilitator was *(circle number)*?**

| **5** (very effective) | **4** | **3** | **2** | **1** (not effective) |
| --- | --- | --- | --- | --- |

**Comments:** …………………………………………………………………………………………………………...

…………………………………………………………………………………………………………...

**9. Would you recommend session to a friend / relative?**

Yes No

**10. Any other comments or suggestions:**

……………………………………………………………………………………………………………

…………………………………………………………………………………………………………...

……………………………………………………………………………………………………………
