# Supplementary material for: Understanding the implementation and effectiveness of a group-based early parenting intervention: a process evaluation protocol
Source: BMC Health Serv Res. 2016 Sep 15;16:490. doi: 10.1186/s12913-016-1737-3 (PMC5025622; doi:10.1186/s12913-016-1737-3)
Supplement: Additional file 2: — The Incredible Years Parent and Baby Program Parent Satisfaction Questionnaire. (PDF 402 kb) [file 12913_2016_1737_MOESM2_ESM.pdf]

Incredible Years®
Parent Program Satisfaction Questionnaire
Babies Program

(Hand out at end of the program)

Participant's Name ________________________________ Date______________________________

The following questionnaire is part of our evaluation of the Incredible Years parenting program that you have received. It is important that you answer as honestly as possible. The information obtained will help us to evaluate and continually improve the program we offer. Your cooperation is greatly appreciated. All responses will be strictly confidential.

A. The Overall Program

Please circle the response that best expresses how you honestly feel at this point.

1. The bonding that I feel with my baby since I took this program is

considerably worse	worse	slightly worse	the same	slightly improved	improved	greatly improved

2. My baby's bonding with me since I started this program is

considerably worse	worse	slightly worse	the same	slightly improved	improved	greatly improved

3. My feelings about my baby's social, emotional and physical developmental progress are that I am

very dissatisfied	dissatisfied	slightly dissatisfied	neutral	slightly satisfied	satisfied	greatly satisfied

4. To what degree has the Incredible Years parenting program helped with other personal or family problems not directly related to your baby (for example, your feelings of support in general)?

hindered much more than helped	hindered	hindered slightly	neither helped nor hindered	helped slightly	helped	helped very much

5. My expectation for good results from the Incredible Years baby program is

very pessimistic	pessimistic	slightly pessimistic	neutral	slightly optimistic	optimistic	very optimistic

6. I feel that the approaches used to enhance my baby's development and language in this program are

very inappropriate	inappropriate	slightly inappropriate	neutral	slightly appropriate	appropriate	greatly appropriate

7. Would you recommend the Incredible Years program to a friend or relative with a baby?

strongly not recommend	not recommend	slightly not recommend	neutral	slightly recommend	recommend	strongly recommend

8. How confident are you in parenting your baby at this time?

very unconfident	unconfident	slightly unconfident	neutral	slightly confident	confident	very confident

9. How confident are you in your ability to provide phyiscal, tacticle and visual stimulation at this time?

very unconfident	unconfident	slightly unconfident	neutral	slightly confident	confident	very confident

10. My overall feeling about achieving my goals in this program for my baby are

very negative	negative	slightly negative	neutral	slightly positive	positive	very positive

B. Incredible Years Teaching Format/Methods

Usefulness

In this section, we would like you to indicate how useful each of the following types of methods used to deliver this program is for you *now*. Please circle the response that most clearly describes your opinion.

1. Content of information presented was

extremely useless	useless	slightly useless	neutral	somewhat useful	useful	extremely useful

2. Demonstration of parenting skills through the use of video vignettes was

extremely useless	useless	slightly useless	neutral	somewhat useful	useful	extremely useful

3. Group discussion, sharing and support from other parents during this program was

extremely useless	useless	slightly useless	neutral	somewhat useful	useful	extremely useful

4. Use of practice with my baby during group sessions was

extremely useless	useless	slightly useless	neutral	somewhat useful	useful	extremely useful

5. I found the “buddy calls” to be

extremely	useless	slightly	neutral	somewhat	useful	extremely
useless		useless		useful		useful

6. The “baby-proof safety checklist” and “things I can do” journal was

extremely	useless	slightly	neutral	somewhat	useful	extremely
useless		useless		useful		useful

7. Practicing things I learned at home with my baby was

extremely	useless	slightly	neutral	somewhat	useful	extremely
useless		useless		useful		useful

8. Weekly handouts (e.g., refrigerator notes) were

extremely	useless	slightly	neutral	somewhat	useful	extremely
useless		useless		useful		useful

9. Phone calls from the group leaders were

extremely	useless	slightly	neutral	somewhat	useful	extremely
useless		useless		useful		useful

C. Specific Parenting Techniques/Topics

Usefulness

In this section, we would like you to indicate how useful each of the following topics and techniques is in improving your interactions with your baby. Please circle the response that most accurately describes the usefulness of the content or techniques.

1. Information about baby’s development and developmental milestones

extremely	useless	slightly	neutral	somewhat	useful	extremely
useless		useless		useful		useful

2. Providing Physical, Tactile and Visual Stimulation (e.g., baby massage, games, exercises)

extremely	useless	slightly	neutral	somewhat	useful	extremely
useless		useless		useful		useful

3. Promoting Baby Language and Brain Development (e.g, speaking “parent-ese”)

extremely	useless	slightly	neutral	somewhat	useful	extremely
useless		useless		useful		useful

4. Child-Directed Play Interactions (e.g., reading babies' cues)

extremely	useless	slightly	neutral	somewhat	useful	extremely
useless		useless		useful		useful

5. Descriptive Commenting/Social and Emotion Coaching

extremely	useless	slightly	neutral	somewhat	useful	extremely
useless		useless		useful		useful

6. Helping Babies Feel Loved, Safe and Secure

extremely	useless	slightly	neutral	somewhat	useful	extremely
useless		useless		useful		useful

7. Singing to Babies

extremely	useless	slightly	neutral	somewhat	useful	extremely
useless		useless		useful		usefu

8. Flexibility in Routines and Transition to Predictable Daily Schedules

extremely	useless	slightly	neutral	somewhat	useful	extremely
useless		useless		useful		useful

9.Gaining Support and Importance of Parental Self-Care

extremely	useless	slightly	neutral	somewhat	useful	extremely
useless		useless		useful		usefu

10. Knowing How to Respond to a Baby's Crying & Strategies for Staying Calm

extremely	useless	slightly	neutral	somewhat	useful	extremely
useless		useless		useful		usefu

11. Introducing Books to Babies

extremely	useless	slightly	neutral	somewhat	useful	extremely
useless		useless		useful		usefu

12.Assuring a Baby-proofed Home

extremely	useless	slightly	neutral	somewhat	useful	extremely
useless		useless		useful		useful

13. This Overall Group of Techniques

extremely	useless	slightly	neutral	somewhat	useful	extremely
useless		useless		useful		useful

D. Evaluation of Incredible Years Parent Group Leader(s)

In this section we would like you to express your opinions about your group leader(s). Please circle the response to each question that best describes how you feel.

Group Leader #1____

(name)

1. I feel that the group leader's teaching and facilitation of group discussions was

very poor	poor	below average	average	above average	superior	excellent

2. The group leader's preparation was

very poor	poor	below average	average	above average	superior	excellent

3. Concerning the group leader's interest and concern in me and my baby, I was

very dissatisfied	dissatisfied	slightly dissatisfied	average	slightly satisfied	satisfied	extremely satisfied

4. At this point, I feel that the group leader in the program was

extremely unhelpful	unhelpful	slightly unhelpful	neutral	slightly helpful	helpful	extremely helpful

If more than one group leader was involved in your program, please fill in the following. (Go to Section E if only one leader was involved.)

Group Leader #2____

(name)

1. I feel that the group leader's teaching and facilitation of group discussion was

very poor	poor	below average	average	above average	superior	excellent

2. The group leader's preparation was

very poor	poor	below average	average	above average	superior	excellent

3. Concerning the group leader's interest and concern in me and my baby, I was

very dissatisfied	dissatisfied	slightly dissatisfied	average	slightly satisfied	satisfied	extremely satisfied

4. At this point, I feel that the group leader in the program was

extremely unhelpful	unhelpful	slightly unhelpful	neutral	slightly helpful	helpful	extremely helpful

E. Overall Program Evaluation

1. What part of the program was most helpful to you?

2. What did you like most about the program?

3. What did you like least about the program?

4. How could the program have been improved to help you more?
