# Supplementary material for: Understanding the implementation and effectiveness of a group-based early parenting intervention: a process evaluation protocol
Source: BMC Health Serv Res. 2016 Sep 15;16:490. doi: 10.1186/s12913-016-1737-3 (PMC5025622; doi:10.1186/s12913-016-1737-3)
Supplement: Additional file 3: — The Incredible Years Parent and Toddler Program Parent Satisfaction Questionnaire. (PDF 54 kb) [file 12913_2016_1737_MOESM3_ESM.pdf]

Incredible Years®
Parent Program Satisfaction Questionnaire
Toddlers Program

(Hand out at end of the program)

Participant's Name ________________________________ Date______________________________

The following questionnaire is part of our evaluation of the Incredible Years parenting program that you have received. It is important that you answer as honestly as possible. The information obtained will help us to evaluate and continually improve the program we offer. Your cooperation is greatly appreciated. All responses will be strictly confidential.

A. The Overall Program

Please circle the response that best expresses how you honestly feel at this point.

1. The bonding that I feel with my baby/toddler since I took this program is

considerably worse	worse	slightly worse	the same	slightly improved	improved	greatly improved

2. My baby/toddler's bonding with me using the methods presented in this program is

considerably worse	worse	slightly worse	the same	slightly improved	improved	greatly improved

3. My feelings about my baby/toddler's social, emotional and physical developmental progress are that I am

very dissatisfied	dissatisfied	slightly dissatisfied	neutral	slightly satisfied	satisfied	greatly satisfied

4. To what degree has the Incredible Years parenting program helped with other personal or family problems not directly related to your child (for example, your marriage, your feelings of support in general)?

hindered much more than helped	hindered	hindered slightly	neither helped nor hindered	helped slightly	helped	helped very much

5. My expectation for good results from the Incredible Years parent program is

very pessimistic	pessimistic	slightly pessimistic	neutral	slightly optimistic	optimistic	very optimistic

6. I feel that the approach used to enhance my baby's/toddler's development in this program is

very inappropriate	inappropriate	slightly inappropriate	neutral	slightly appropriate	appropriate	greatly appropriate

7. Would you recommend the program to a friend or relative?

strongly not recommend	not recommend	slightly not recommend	neutral	slightly recommend	recommend	strongly recommend

8. How confident are you in parenting at this time?

very unconfident	unconfident	slightly unconfident	neutral	slightly confident	confident	very confident

9. How confident are you in your ability to manage *future* behavior problems in the home using what you learned from this program?

very unconfident	unconfident	slightly unconfident	neutral	slightly confident	confident	very confident

10. My overall feeling about achieving my goals in this program for my child and family are

very negative	negative	slightly negative	neutral	slightly positive	positive	very positive

B. Teaching Format

Usefulness

In this section, we would like you to indicate how useful each of the following types of teaching is for you *now*. Please circle the response that most clearly describes your opinion.

1. Content of information presented was

extremely useless	useless	slightly useless	neutral	somewhat useful	useful	extremely useful

2. Demonstration of parenting skills through the use of video vignettes was

extremely useless	useless	slightly useless	neutral	somewhat useful	useful	extremely useful

3. Group discussion of parenting skills was

extremely useless	useless	slightly useless	neutral	somewhat useful	useful	extremely useful

4. Use of practice/role play during group sessions was

extremely useless	useless	slightly useless	neutral	somewhat useful	useful	extremely useful

5. I found the "buddy calls" to be

extremely useless	useless	slightly useless	neutral	somewhat useful	useful	extremely useful

6. Reading chapters from the Incredible Years book or listening to the CD was

extremely useless	useless	slightly useless	neutral	somewhat useful	useful	extremely useful

7. Practicing skills at home with my child was

extremely useless	useless	slightly useless	neutral	somewhat useful	useful	extremely useful

8. Weekly handouts (e.g., refrigerator notes) were

extremely useless	useless	slightly useless	neutral	somewhat useful	useful	extremely useful

9. Phone calls from the group leaders were

extremely useless	useless	slightly useless	neutral	somewhat useful	useful	extremely useful

C. Specific Parenting Techniques

Usefulness

In this section, we would like you to indicate how useful each of the following techniques is in improving your interactions with your child and decreasing his or her "inappropriate" behaviors *now*. Please circle the response that most accurately describes the usefulness of the content or skills.

1. Information about infant/toddler development and developmental milestones

extremely useless	useless	slightly useless	neutral	somewhat useful	useful	extremely useful

2. Providing Physical, Tactile and Visual Stimulation

extremely useless	useless	slightly useless	neutral	somewhat useful	useful	extremely useful

3. Promoting Infant and Toddler Language Development

extremely useless	useless	slightly useless	neutral	somewhat useful	useful	extremely useful

4. Child-Directed Play

extremely useless	useless	slightly useless	neutral	somewhat useful	useful	extremely useful

5. Descriptive Commenting/Social, Emotion and Academic Coaching

extremely useless	useless	slightly useless	neutral	somewhat useful	useful	extremely useful

6. Praise and Encouragement

extremely useless	useless	slightly useless	neutral	somewhat useful	useful	extremely useful

7. Spontaneous Rewards

extremely useless	useless	slightly useless	neutral	somewhat useful	useful	extremely useful

8. Routines, Separation & Reunions

extremely useless	useless	slightly useless	neutral	somewhat useful	useful	extremely useful

9. Ignoring

extremely useless	useless	slightly useless	neutral	somewhat useful	useful	extremely useful

10. Positive Discipline

extremely useless	useless	slightly useless	neutral	somewhat useful	useful	extremely useful

11. Information about baby- and toddler-proofing at home

extremely useless	useless	slightly useless	neutral	somewhat useful	useful	extremely useful

12. This Overall Group of Techniques

extremely useless	useless	slightly useless	neutral	somewhat useful	useful	extremely useful

D. Evaluation of Parent Group Leader(s)

In this section we would like you to express your opinions about your group leader(s). Please circle the response to each question that best describes how you feel.

Group Leader #1____

(name)

1. I feel that the group leader's teaching was

very poor	poor	below average	average	above average	superior	excellent

2. The group leader's preparation was

very poor	poor	below average	average	above average	superior	excellent

3. Concerning the group leader's interest and concern in me and my child, I was

very dissatisfied	dissatisfied	slightly dissatisfied	average	slightly satisfied	satisfied	extremely satisfied

4. At this point, I feel that the group leader in the program was

extremely unhelpful	unhelpful	slightly unhelpful	neutral	slightly helpful	helpful	extremely helpful

5. Concerning my personal feelings toward the group leader, I

dislike him/her very much	dislike him/her	dislike him/her slightly	have a neutral attitude toward him/her	like him/her slightly	like him/her	like him/her very much

If more than one group leader was involved in your program, please fill in the following. (Go to Section E if only one leader was involved.)

Group Leader #2____

(name)

1. I feel that the group leader's teaching was

very poor	poor	below average	average	above average	superior	excellent

2. The group leader's preparation was

very poor	poor	below average	average	above average	superior	excellent

3. Concerning the group leader's interest and concern in me and my child, I was

very dissatisfied	dissatisfied	slightly dissatisfied	average	slightly satisfied	satisfied	extremely satisfied

4. At this point, I feel that the group leader in the program was

extremely unhelpful	unhelpful	slightly unhelpful	neutral	slightly helpful	helpful	extremely helpful

5. Concerning my personal feelings toward the group leader, I

dislike him/her very much	dislike him/her	dislike him/her slightly	have a neutral attitude toward him/her	like him/her slightly	like him/her	like him/her very much

E. Overall Program Evaluation

1. What part of the program was most helpful to you?

2. What did you like most about the program?

3. What did you like least about the program?

4. How could the program have been improved to help you more?
