# Supplementary material for: Understanding the implementation and effectiveness of a group-based early parenting intervention: a process evaluation protocol
Source: BMC Health Serv Res. 2016 Sep 15;16:490. doi: 10.1186/s12913-016-1737-3 (PMC5025622; doi:10.1186/s12913-016-1737-3)
Supplement: Additional file 4: — Example implementer/facilitator feedback form. (DOCX 13 kb) [file 12913_2016_1737_MOESM4_ESM.docx]

**Sample facilitator feedback form**

**Name: ___________________________________________________________**

**Date: ____________________________________________________________**

**Which programmes/workshops were you involved in delivering?**

**What are the core ‘topics’ (e.g. information, skills, strategies) involved in the workshop?**

**______________________________________________________________________________________________________________________________________________________________________________________________________________________________________________________________________________________________________________________________________________________________________________________________________________________________________________________________________________________________________________________________**

**What percentage of these ‘topics’ did you cover, on average, in the workshop?**

**Cycle 1:**

Less than 25% 25-50% 51%-75% 76%-90% 91-100%

**How often did you prepare with colleagues/co-facilitators for delivery?**

Not at all Very little Some Quite a bit Extensive

**Did you complete a checklists after each group session?**

Not at all Very little Some Quite a bit Extensive

**How often did you….**

*Write an agenda on the board?* Not at all Very little Some Quite a bit Extensive

*Review group ground rules?* Not at all Very little Some Quite a bit Extensive

*Provide parents with hand outs?* Not at all Very little Some Quite a bit Extensive

*Assign home activities?*  Not at all Very little Some Quite a bit Extensive

**How often do you engage in self-evaluation after sessions?**

Not at all Very little Some Quite a bit Extensive

**How often did you call or contact parents after your sessions?**

Not at all Very little Some Quite a bit Extensive

**On average, how easy or difficult was it to engage parents in each weekly session?**

Very easy Somewhat easy Neither easy or difficult Somewhat difficult Very difficult

**Were parents supporting each other during the session?**

Not at all Very little Some Quite a bit Extensive
