# Supplementary material for: Understanding the implementation and effectiveness of a group-based early parenting intervention: a process evaluation protocol
Source: BMC Health Serv Res. 2016 Sep 15;16:490. doi: 10.1186/s12913-016-1737-3 (PMC5025622; doi:10.1186/s12913-016-1737-3)
Supplement: Additional file 5: — Example stakeholder survey questionnaire. (DOCX 21 kb) [file 12913_2016_1737_MOESM5_ESM.docx]

**Instructions**

**You have received this questionnaire because you play a very important role in the implementation and delivery of the Parent and Infant programme, so we would like to ask you some questions about your thoughts and opinions of the Parent and Infant programme.**

**There are no right or wrong responses and all of the information you provide is completely anonymous and confidential, so please be as honest as possible.**

**Personal information**

**Work role: _________________________________________________**

**Background**

**1. With which aspect(s) of the Parent and Infant programme are you currently involved?**

***______________________________________________________________________________________________________________________________________________________________________________________________________________________________________________________***

**2. To what extent do you think that your organisation is an important part of the work of Parent and Infant programme?**

| 1 | 2 | 3 | 4 | 5 |
| --- | --- | --- | --- | --- |
| Very unimportant | Unimportant | Neither important nor unimportant | Important | Very important |

**3. To what extent do you think that the Parent and Infant programme is an important part of the work of your organisation?**

| 1 | 2 | 3 | 4 | 5 |
| --- | --- | --- | --- | --- |
| Very unimportant | Unimportant | Neither important nor unimportant | Important | Very important |

**Capacity building and programme support**

**4. What training/capacity building services have been/are available to you OR your organisation to support your involvement in the Parent and Infant programme?**

***____________________________________________________________________________________________________________________________________________________________________________________________________________________________________________________________________________________________________________________________________________________________________________________________________________________________________________________________________________________________________________***

**5. How satisfied are you with these training/capacity building services?**

| 1 | 2 | 3 | 4 | 5 |
| --- | --- | --- | --- | --- |
| Highly dissatisfied | Dissatisfied | Neither satisfied nor dissatisfied | Satisfied | Highly satisfied |

**6. Have specific targets (e.g. service uptake; parent outcomes; child outcomes) been set for the Parent and Infant programme?**

| Yes | No | Unsure |
| --- | --- | --- |

**7. If ‘yes’, to what extent will those targets been reached (in the specified time-scale)?**

Definitely won’t Probably won’t Probably will Definitely will

**8. Have clear plans and/or protocols been put in place to guide programme delivery and/or implementation fidelity?**

| 1 | 2 | 3 | 4 | 5 |
| --- | --- | --- | --- | --- |
| Strongly disagree | Disagree | Neither agree nor disagree | Agree | Strongly disagree |

**9. Have clear plans and/or protocols been put in place to regularly monitor and assess the quality of programme delivery and/or implementation fidelity?**

| 1 | 2 | 3 | 4 | 5 |
| --- | --- | --- | --- | --- |
| Strongly disagree | Disagree | Neither agree nor disagree | Agree | Strongly disagree |

**10. Is sufficient work time available for you to attend meetings regarding the implementation of the Parent and Infant programme?**

| 1 | 2 | 3 | 4 | 5 |
| --- | --- | --- | --- | --- |
| Strongly disagree | Disagree | Neither agree nor disagree | Agree | Strongly disagree |

**11. Is there a clear management structure in place with responsibility for overseeing that the Parent and Infant programme is delivered in accordance with plans/protocols?**

| 1 | 2 | 3 | 4 | 5 |
| --- | --- | --- | --- | --- |
| Strongly disagree | Disagree | Neither agree nor disagree | Agree | Strongly disagree |

**Programme implementation**

**12. Do you feel that clear and stable referral procedures and protocols are in place for the Parent and Infant programme?**

| 1 | 2 | 3 | 4 | 5 |
| --- | --- | --- | --- | --- |
| Strongly disagree | Disagree | Neither agree nor disagree | Agree | Strongly disagree |

**13. Is sufficient work time available to refer parents to participate in the Parent and Infant programme?**

| 1 | 2 | 3 | 4 | 5 |
| --- | --- | --- | --- | --- |
| Strongly disagree | Disagree | Neither agree nor disagree | Agree | Strongly disagree |

**14. Is sufficient work time available to refer ‘hard to reach’ parents to participate in the Parent and Infant programme?**

| 1 | 2 | 3 | 4 | 5 |
| --- | --- | --- | --- | --- |
| Strongly disagree | Disagree | Neither agree nor disagree | Agree | Strongly disagree |

**15. Are sufficient resources (e.g. financial) available to refer parents to participate in the programme?**

| 1 | 2 | 3 | 4 | 5 |
| --- | --- | --- | --- | --- |
| Strongly disagree | Disagree | Neither agree nor disagree | Agree | Strongly disagree |

**16. Is satisfactory space available to deliver workshops and/or IY programmes as part of the Parent and Infant programme?**

| 1 | 2 | 3 | 4 | 5 |
| --- | --- | --- | --- | --- |
| Strongly disagree | Disagree | Neither agree nor disagree | Agree | Strongly disagree |

**17. Are sufficient resources (e.g. financial; personnel; handouts or other materials) available to support delivery of IY group sessions/workshops?**

| 1 | 2 | 3 | 4 | 5 |
| --- | --- | --- | --- | --- |
| Strongly disagree | Disagree | Neither agree nor disagree | Agree | Strongly disagree |

**18. Is there sufficient administrative assistance and support available to help with the delivery of IY group sessions/workshop?**

| 1 | 2 | 3 | 4 | 5 |
| --- | --- | --- | --- | --- |
| Strongly disagree | Disagree | Neither agree nor disagree | Agree | Strongly disagree |

**19. Is sufficient work time available for you/your organisation to maintain contact/follow-up with parents after the delivery of IY group sessions/workshops?**

| 1 | 2 | 3 | 4 | 5 |
| --- | --- | --- | --- | --- |
| Strongly disagree | Disagree | Neither agree nor disagree | Agree | Strongly disagree |

**20. Is there currently a clear plan in place for the future delivery of the Parent and Infant programme?**

| 1 | 2 | 3 | 4 | 5 |
| --- | --- | --- | --- | --- |
| Strongly disagree | Disagree | Neither agree nor disagree | Agree | Strongly disagree |

**Programme Perceptions**

**21. To what degree to you feel your involvement in the Parent and Infant programme has helped you with other aspects of your work with parents, children and/or families?**

| Not at all | Not much | Some | Quite a lot | A great deal |
| --- | --- | --- | --- | --- |
|  |  |  |  |  |

**22. I would describe the impact of my participation in the Parent and Infant programme on my day-to-day work as...**

| Very negative | Negative | Neutral | Positive | Very positive |
| --- | --- | --- | --- | --- |
|  |  |  |  |  |

**23. My expectations for good outcomes for parents from the Parent and Infant programme is....**

| Very negative | Negative | Neutral | Positive | Very positive |
| --- | --- | --- | --- | --- |
|  |  |  |  |  |

**24. My expectations for good outcomes for infants from the Parent and Infant programme is.....**

| Very negative | Negative | Neutral | Positive | Very positive |
| --- | --- | --- | --- | --- |
|  |  |  |  |  |

**25. Please rate the extent to which you agree or disagree with the following statements:**

***Participating in the delivery of the Parent and Infant has helped improve my service’s/ organisation’s work with children, young people and/or parents in the area:***

| 1 | 2 | 3 | 4 | 5 |
| --- | --- | --- | --- | --- |
| Strongly disagree | Disagree | Neither agree nor disagree | Agree | Strongly disagree |

***Participating in the delivery of the Parent and Infant has helped improve communication between organisations working with children, young people and parents in the area:***

| 1 | 2 | 3 | 4 | 5 |
| --- | --- | --- | --- | --- |
| Strongly disagree | Disagree | Neither agree nor disagree | Agree | Strongly disagree |

***Participating in the delivery of the Parent and Infant has helped to improve collaboration between organisations working with children, young people and parents in the area:***

| 1 | 2 | 3 | 4 | 5 |
| --- | --- | --- | --- | --- |
| Strongly disagree | Disagree | Neither agree nor disagree | Agree | Strongly disagree |

***Participating in the delivery of the Parent and Infant has helped to build closer working relationships between organisations working with children, young people and parents in the area:***

| 1 | 2 | 3 | 4 | 5 |
| --- | --- | --- | --- | --- |
| Strongly disagree | Disagree | Neither agree nor disagree | Agree | Strongly disagree |

**26. What are your goals in relation to taking part in the Parent and Infant programme?**

***1.________________________________________________________________________________ __________________________________________________________________________________2.________________________________________________________________________________ __________________________________________________________________________________3.__________________________________________________________________________________________________________________________________________________________________***

**27. What are your expectations for taking part in the Parent and Infant programme?**

***1.________________________________________________________________________________ __________________________________________________________________________________2.________________________________________________________________________________ __________________________________________________________________________________3.__________________________________________________________________________________________________________________________________________________________________***
