# Supplementary material for: Understanding the implementation and effectiveness of a group-based early parenting intervention: a process evaluation protocol
Source: BMC Health Serv Res. 2016 Sep 15;16:490. doi: 10.1186/s12913-016-1737-3 (PMC5025622; doi:10.1186/s12913-016-1737-3)
Supplement: Additional file 6: — Example qualitative interview schedule with parents. (DOCX 15 kb) [file 12913_2016_1737_MOESM6_ESM.docx]

**Interview schedule with parents**

**Background**

**Personal**

- Just to get started, could you tell me a little bit about yourself?
- Would you say that having your baby has changed your sense of self? Or how you feel about yourself?
  - Were there positive/negative changes?

**Background and parenting experiences**

- How prepared did you feel to handle the challenges of parenthood?
  - Do you have past experience with young children/other children?
- Could you tell me about your experiences of getting to know your baby over the past few weeks?
- Do you have any insecurities about “how to be” a parent? Could you tell me a little more about that?
  - What has been the biggest challenge you have faced?
  - What do you feel are the main challenges of parenting?
- Looking back at the first few months of baby’s life, is there anything that you would change or like to have done differently?

**Home life, relationships and lifestyle**

- How did having a baby affect your way of managing at home?
  - How do ye as a family manage the household and baby care/childcare jobs?
- Have you found it more difficult to find time together as a couple?
  - How has that affected your relationship (Were there positive or negative changes to your relationship)?

**Experiences of Parent and Infant Programme**

- Can you tell me about your experience of the Parent and Infant Programme?
- What did you like best about the programme?
- What did you like least about the programme?

**Parenting experiences**

- How would you describe your relationship with your baby now?
  - Of course, your baby is constantly changing and growing, to what degree do you feel the programme helped you to deal with the changing needs of your baby?
- To what extent do you feel that the programme changed your parenting?
- Were any particular parts of the programme especially useful in handling the challenges of being a parent?
  - Were any parts of the programme less valuable?
- To what degree has being involved in the programme helped with other personal or family issues
  - (e.g. sharing the household and baby care/childcare jobs?)
  - What about your time together as a couple?

(If applicable)

- Could you tell me a little bit about how you experience your work life?
  - What has changed for you in your work role? Do you think differently about your work? To what extent has the programme been beneficial for you in relation to your work commitments?
- What changes have you noticed in yourself since taking part in the programme? Has anything changed for you around how you see yourself as a parent?
  - Positive/negative outcomes?
- What are your expectations now for the future?

**Further detail**

- Overall, what did you think about content of the Parent and Infant programme? What about the way the programme was delivered (what did you think about the group leaders/facilitators; videos, vignettes, materials)?
- To what extent did you find the group supportive?
- Did you find the baby massage useful? What about the other workshops?
- Were there any parts of the programme that you had any difficulty with, or that you felt challenged the way you feel as a parent?
- Overall, is there anything you would change about the programme?
- Did you find it easy to make the sessions? The workshops?

**Finishing up**

- If you met another parent who had just had a baby and was thinking of taking part in the Parent and Infant programme, what would you say to them now?
- To what extent do you think that the programme should be available to other parents throughout the country?
- Do you have anything else you would like to add?
