# Supplementary material for: Understanding the implementation and effectiveness of a group-based early parenting intervention: a process evaluation protocol
Source: BMC Health Serv Res. 2016 Sep 15;16:490. doi: 10.1186/s12913-016-1737-3 (PMC5025622; doi:10.1186/s12913-016-1737-3)
Supplement: Additional file 7: — Example qualitative interview schedule with implementers. (DOCX 16 kb) [file 12913_2016_1737_MOESM7_ESM.docx]

**Background**

- So just to get started then could you give me a brief overview of your background/ work role?
- Could you tell me a little more about your role vis-a-vis the Parent and Infant programme?
- Could you tell me a little about the culture within your organisation?
- How does this relate to your organisation’s involvement with the Parent and Infant programme?

**Overview of the Parent and Infant programme**

- Could you give me your understanding of the Parent and Infant programme and its different elements?
- How do you understand the purpose and aims of the Parent and Infant programme?
- How has the Parent and Infant programme changed services and service provisions for parents and infants?
- What do you see as the key outcomes for parents and infants?

How will those key outcomes be achieved? What ingredients are necessary to achieve those outcomes?

- - Could you tell me about the referral procedures that are in place to recruit parents to the Parent and Infant programme?
- In your opinion, are those referral procedures working? Are they stable over the longer term?
- How could recruitment of parents be improved, if necessary?
- The Parent and Infant Programme is a longitudinal programme, so it offers services and supports for 2 years – How do you see that process unfolding?
- In your opinion, how important is it for parents to participant for the full cycle?
- What resources and supports are necessary to encourage parents to stay with the programme?
- Are their barriers to that engagement being achieved?
- One of the challenges with these types of programmes is engaging hard to reach parents, how do you deal with this issue in relation to the Parent and Infant programme?
- Is there anything that you would like to see put in place to support the engagement of harder to reach families?

**Implementation of the Parent and Infant programme**

- You play an important role in the delivery/implementation of the Up to 2 programme, how is your involvement supported within your organisation?
- Are there any difficulties in maintaining your involvement?
- Could you tell me a little about your experience of running the workshop/parenting programme?
  - What are the main issues or challenges that arise for you in running the workshop/parenting programme?
- How has the experience of running the workshop/parenting programme changed over time?
  - Which elements of the workshop/parenting programme do you think work best for parents?
  - What elements of the workshop/parenting programme do you think are less useful for parents?
  - How would you describe your relationship with parents?
  - Have you experienced any issues that impact on how parents fare during the programme? (e.g. group size; socioeconomic group)
  - How do group differences impact on how parents fare on the programme? (e.g. first time mothers, second time mothers; disadvantage)
- What do you see as the key inputs, mechanisms or resources that ensure the effective implementation of the Parent and Infant programme?
- How do you see implementation proceeding at the moment?
- For you, what are the most important ingredients involved in making the Parent and Infant programme work?
- What are the barriers/threats to effective implementation?
- What are the potential challenges to the long-run implementation of the programme?
- The Parent and Infant Programme involves a broad range of different stakeholders, could you tell me who the key stakeholders are in the implementation of the Parent and Infant programme?
- How do those relationships support the implementation of the programme?
- How are those relationships working?
- Is there anything that could undermine the participation of stakeholders? Are those relationships sustainable?

**Summing up**

- Is there anything that you would change about the Parent and Infant programme?
- Is there anything else you would like to add?
